# HealthWorks: results of a multi-component group-randomized worksite environmental intervention trial for weight gain prevention

**DOI:** 10.1186/1479-5868-9-14

**Published:** 2012-02-16

**Authors:** Jennifer A Linde, Katherine E Nygaard, Richard F MacLehose, Nathan R Mitchell, Lisa J Harnack, Julie M Cousins, Daniel J Graham, Robert W Jeffery

**Affiliations:** 1 Division of Epidemiology & Community Health, School of Public Health, University of Minnesota, Minneapolis, MN, USA; 2 Division of Biostatistics, School of Public Health, University of Minnesota, Minneapolis, MN, USA; 3 School of Kinesiology, College of Education and Human Development, University of Minnesota, Minneapolis, MN, USA; 4 Division of Epidemiology & Community Health, School of Public Health, University of Minnesota, 1300 S. 2nd Street, Suite 300, Minneapolis, MN 55454-1015, USA

**Keywords:** Obesity, Worksites, Adults, Environment, Weight gain prevention

## Abstract

**Background:**

U.S. adults are at unprecedented risk of becoming overweight or obese, and most scientists believe the primary cause is an obesogenic environment. Worksites provide an opportunity to shape the environments of adults to reduce obesity risk. The goal of this group-randomized trial was to implement a four-component environmental intervention at the worksite level to positively influence weight gain among employees over a two-year period. Environmental components focused on food availability and price, physical activity promotion, scale access, and media enhancements.

**Methods:**

Six worksites in a U.S. metropolitan area were recruited and randomized in pairs at the worksite level to either a two-year intervention or a no-contact control. Evaluations at baseline and two years included: 1) measured height and weight; 2) online surveys of individual dietary intake and physical activity behaviors; and 3) detailed worksite environment assessment.

**Results:**

Mean participant age was 42.9 years (range 18-75), 62.6% were women, 68.5% were married or cohabiting, 88.6% were white, 2.1% Hispanic. Mean baseline BMI was 28.5 kg/m^2 ^(range 16.9-61.2 kg/m^2^). A majority of intervention components were successfully implemented. However, there were no differences between sites in the key outcome of weight change over the two-year study period (*p *= .36).

**Conclusions:**

Body mass was not significantly affected by environmental changes implemented for the trial. Results raise questions about whether environmental change at worksites is sufficient for population weight gain prevention.

**Trial Registration:**

ClinicalTrials.gov: NCT00708461

## Background

Approximately 140 million U.S. adults (60% of the free-living population) are employed [1]. Recent statistics indicate that 68% of U.S. adults are overweight, with 33% classified as obese [2]. Adults are at continued risk for weight gain over time, with average increases of two pounds per year [3]. Obesity is rising across all employment groups, with no differences in increases by race/ethnicity or sex [4]. Obesity is associated with negative consequences in working populations, including more frequent absenteeism, sick leave [5,6] or workplace injury and disability pension claims [7,8], and greater health care costs [5].

As employed adults spend approximately half of waking time at work [9], worksites provide a logical setting in which the environment might be reshaped to promote healthier behaviors and improve weight control. Employers may be motivated to make changes due to concerns about quality or cost of employee health care [10], and worksites may be in a unique position to leverage resources (e.g. food service, communication networks, Human Resources departments) and promote social support among co-workers for obesity-preventive behaviors.

Environmental changes that make conceptual sense for obesity prevention include targeting food service (i.e. availability of energy-dense foods, portion sizes, cost), the physical environment (e.g. opportunities for exercise), and information distributed to increase knowledge of behaviors related to obesity risk [3,11]. Previous studies designed to address obesity in the workplace typically have focused on a single factor from this list of options or had other limitations to the study design that preclude systematic examination of the effects of environmental changes on body weight over a prolonged period of time.

With regard to promoting changes to the food environment, one study focused on availability of healthy foods in cafeterias and was successful in reducing prices by 50% and increasing purchasing of fruit and salad by 300%, but was conducted in only a single worksite over a three-week period, using a pre-post observational design rather than randomizing to intervention or control conditions, and did not assess body weight as part of the study [12]. Another study targeted pricing and promotion of healthy vending machine snacks in worksites and schools over a one-year period and achieved significant increases in purchases of healthy snacks by lowering prices by 50% and enhancing promotion; however, as with the study noted above, body weight was not monitored as part of this trial, so the effects on weight outcomes over time are not known [13].

Other studies have focused on aspects of the environment that lend themselves to physical activity. A 2004 study examined pedometer use among 177 sedentary employees using a 12-week pre-post design; 59% of employees completed the program, which resulted in an average increase of over 3,400 steps per day from baseline and a small but statistically significant change in BMI; this study is limited by a relatively small sample size, short study duration, and lack of randomization to conditions [14]. Stair use at work has also been targeted for intervention. In one study, the Centers for Disease Control and Prevention in the United States used one of their office buildings to test the effects of stairwell enhancements (updated carpet and paint, artwork, music, and motivational signs throughout the building) on employee stair use assessed over a 3.5-year period [15]. Stair use, measured objectively using infrared proximity sensors, increased by 4.7% overall; however, this study did not include a comparison condition without stair intervention, nor did it assess body weight or track changes in weight over time [15].

In addition to the limited focus or methodological limitations of the trials noted above, most studies in this area have focused on behavioral and informational changes in worksites rather than strictly environmental or policy-related changes [16]. For example, an earlier trial by the present research team investigated the effects of a program offering weight control or smoking cessation classes four times in two years at 16 worksites randomized to intervention, versus 16 sites randomized to an assessment-only control condition [17]. The intervention had no significant effects on weight over two years relative to the control condition, likely due to very low participation in weight loss classes during the study (16% of all employees during the first wave, approximately 5% in subsequent waves) and reliance on attracting employees to a separate educational program for weight loss rather than integrating healthy weight-related behaviors into the worksite environment as a whole [18].

To address obesity among working adults, the goal of the HealthWorks group-randomized trial was to implement a multi-component program to alter the worksite environment, with the intent of reducing weight gain [expressed in body mass index (BMI) units] over the study period, relative to control sites with no investigator-initiated alterations to the environment. The results of the first weight-gain prevention trial funded by NIH set the stage for the approach taken in the current trial. The Pound of Prevention (POP) study was conducted from 1995 to 1999 by the present investigators [19]. Its purpose was to evaluate whether weight gain could be prevented in healthy young community-dwelling adults through education. Participants (228 men and 998 women aged 20 through 45 years) were recruited as individuals via telephone contacts, mailed or newspaper advertisements, and face-to-face contacts, and randomized to an intervention consisting principally of a monthly educational newsletter or to a no-treatment control condition. Five behavior changes were advocated: 1) increase frequency of self-weighing, 2) increase physical activity, 3) increase fruit intake, 4) increase vegetable intake, and 5) decrease intake of high-fat foods. After 3 years, the intervention was successful in increasing knowledge, self-weighing frequency, and healthy weight control behaviors [19]. In addition, aggregated across all treatment groups, individuals who reported adopting these study recommendations were more successful in preventing weight gain over time than those who did not [20]. However, the intervention did not significantly slow the rate of weight gain over time in the intervention group when compared to the group receiving no treatment.

Drawing on the literature described above in terms of representative studies, the two main goals of the HealthWorks trial were to a) conduct a simultaneous test of environmental approaches to healthy weight choices (including eating, physical activity, and weight monitoring behaviors), and b) extend the test of these approaches to a more sustained time period than typically observed. The study was conceptualized in terms of social cognitive theory (SCT), which argues that environment, behavior, and cognition are reciprocally related, such that changes to the environment should influence behavior [21]. Intervention efforts primarily focused on the environmental change component due to a relative dearth of studies in that area, as well as being driven by a social ecological interest in taking a broader worksite-level approach to obesity prevention rather than an individualized behavioral approach to the issue [22], with the consideration that such a program has potential to be sustained in worksites at a lower cost than high intensity, individual behavior change efforts.

The four major environmental change components addressed food selection, promotion of walking/stair use, weight self-monitoring, and health information at work; we hypothesized that employees at sites with environmental changes would gain less weight over the two-year study period, relative to those employees at sites with no changes; based on weight gain expectations for an untreated adult population over a two-year period, we expected to observe weight gain of approximately 1 kilogram (kg) per year [3]. These components have been tested separately in previous worksite or other obesity prevention studies and found to be effective in terms of impact on the environment, though most previous trials were of relatively short duration and not all assessed weight changes as an outcome [12-17,23]. The addition of a weight self-monitoring component to the current project addresses an obesity prevention strategy with minimal evaluation in worksites. The current study represents an effort to test these components using an integrated worksite environmental approach, rather than by relying on individual or small-group weight education and health promotion activities [24] or attempts to alter workplace norms related to weight [25]. Knowledge from this trial has the potential to promote a healthier workforce and improve population weight control.

## Methods

### Study design and recruitment

HealthWorks is a group-randomized trial of six worksites in the Twin Cities metropolitan area. Study approval was granted by the University of Minnesota Institutional Review Board. As extensive worksite and participant recruitment and evaluation took place prior to intervention activities, and as evaluation results directly shaped intervention procedures, the recruitment and evaluation procedures will be described first, followed by a description of the randomization and intervention delivery protocols.

#### Site recruitment

Worksite recruitment process information is presented in Table 1. Site recruitment took place between June 2005 and December 2006. Dun & Bradstreet database listings were used to identify 200 potential worksites meeting basic eligibility requirements of site size (250-1,000 employees) and location. Telephone calls were placed to sites to ascertain specific site eligibility characteristics including interest in research participation, presence of food service, a building with at least two floors (to ensure presence of stairs on site), minimal seasonal fluctuation of employees, stability of location and workforce over the next several years, and willingness to provide employees' work contact information. Successful screening calls identified 37 sites as potential study locations; of the remaining 163 sites, approximately half were ineligible, just over one-third could not be reached, and 10% declined. A letter then was sent to human resources personnel (identified in the initial screening call) to provide basic study information and request contact to discuss the study and eligibility requirements further. The next telephone contact confirmed basic site eligibility criteria and explained the purpose and nature of the study. The principal investigator and project director then visited 11 sites for one-hour visits with site executives and human resources staff, to present the study design and aims and field questions from site personnel. Following these visits, six sites agreed to participate in the study, three sites declined, and two sites did not respond to follow-up contact attempts by the project director. The six sites recruited to the study represent 54.5% of all sites that completed an investigator-initiated visit. Recruited sites averaged 450 employees (range = 284-639).

**Table 1:** Worksite recruitment process chart

N	Percent at Screening Stage	Percent of Total	Status
**200**	**100%**	**100%**	**Telephone calls placed to screen for eligibility and obtain name of contact person to receive initial letter**

**163**		**81.5%**	**No letter sent after screening call**
84	51.5%	42%	Ineligible at screening
56	34.4%	28%	No response to screening call
15	9.2%	7.5%	Declined due to lack of interest
8	4.9%	4%	Site contact outcome not recorded

**37**		**18.5%**	**Letter sent after screening call**
25	67.6%	12.5%	Investigator-initiated calls to discuss project
10	27%	5%	Did not respond to investigator call
2	5.4%	1%	Not pursued for study due to company factors (type of company or known merger issues)

**25**		**12.5%**	**Investigator-initiated call to discuss project details**
15	60%	7.5%	Scheduled an in-person or telephone visit
5	20%	2.5%	Declined due to lack of time or interest
3	12%	1.5%	Did not follow up to schedule or declined to participate
2	8%	1%	Not pursued further due to type of company

**15**		**7.5%**	**Site visits (in-person or by telephone)**
11	73.3%	5.5%	Site visit completed
3	20%	1.5%	Site visit postponed; not rescheduled due to completion of recruitment
1	6.7%	0.5%	Site visit no-show (scheduled telephone call); not rescheduled due to completion of recruitment

**11**		**5.5%**	**Recruitment following site visits**
6	54.5%	3%	Successfully recruited
3	27.3%	1.5%	Declined participation after site visit
2	18.2%	1%	No follow-up response to investigator contacts after site visit

#### Participant recruitment

Human resources departments at the six recruited sites provided a database of worksite contact information (email and telephone) for all eligible employees. Employees were considered eligible if they were employed at least 50% time on-site during a daytime shift. Study staff worked with site personnel to distribute a company-wide email announcement, via company email address, to all eligible employees. The message described the partnership with the University and gave a brief description of the study. Employees were notified that a study staff member would contact them about the project within the next two weeks and were given the option to call study staff in advance to opt out or to enroll in the study.

**Figure 1:**
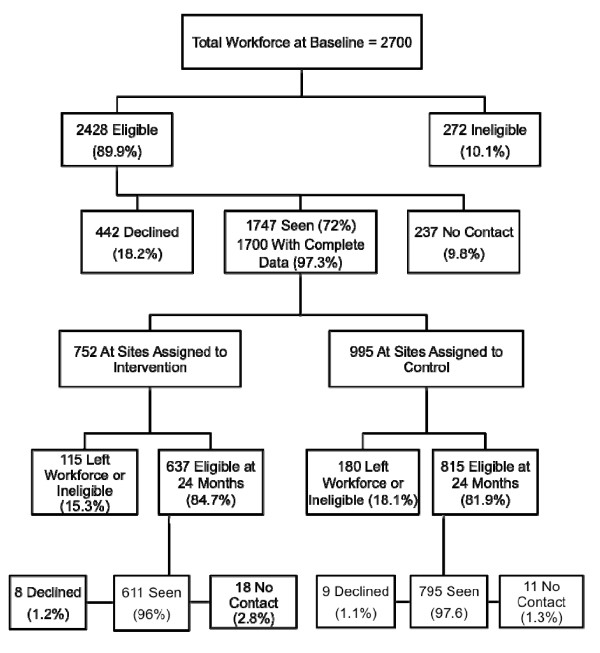
**Individual participant recruitment flow diagram**.

Study evaluation staff contacted employees to discuss the study and invite them to participate in evaluation procedures. Staff followed a protocol of up to nine contact attempts (either by telephone or email) of each employee during the scheduling process. The individual participant recruitment flow diagram is presented in Figure 1.

### Site measures

At baseline and 24 months, key environmental features of each worksite were evaluated by the study team over a one-month period; an abbreviated one-week environmental scan was performed at 12 months. Study staff members were sent to each site to collect data, consulting with worksite employees as needed. Environmental assessments included:

#### Food inventory

Detailed data on foods present in the cafeteria during the lunch meal period and in on-site vending machines were collected by study staff, following procedures implemented in prior comparable studies [13,26,27]. The team recorded information on available foods, including price, portion size, and calories per portion. For packaged foods, portions and calories per package were also recorded. When food product information was not visible on a package, study staff consulted with the on-site food manager or vending supplier (e.g., to obtain recipes for prepared foods such as soups or sandwiches, or to search inventory lists), searched manufacturer websites, or accessed detailed nutrient information via the Nutrition Data System for Research (NDSR, Nutrition Coordinating Center, University of Minnesota, Minneapolis, MN). All food data were collected on standardized record forms and entered into a Filemaker Pro database maintained by the study data manager. At intervention sites, a suggested price (see *Intervention Procedures *below) was recorded in the database as well.

Foods were classified as "calorie smart" based on guidelines from the California School Nutrition Association standards http://www.calsna.org/ for healthy portion sizes, according to the following categories: entrée (e.g., meat, egg, poultry, or mixed dish, large soup, vegetarian meat substitute; ≤ 500 calories), side dish (e.g., small soup, bread, potato, rice, vegetable; ≤ 250 calories), snack (e.g., chips, granola bar, yogurt; ≤ 150 calories), beverage (e.g., coffee, dairy drinks, fruit drinks, soda; ≤ 150 calories), desserts (e.g., cookies, pie, cakes, ice cream, sweet rolls; ≤ 200 calories), condiments (e.g., ketchup, salad dressing; ≤ 40 calories), and combination foods (e.g., packaged meals consisting of entrée and side dish, with or without beverage, for a reduced price relative to a la carte purchase of the same food products; ≤ 750 calories). Baseline food data were used to determine targets for healthy food pricing and availability at intervention sites.

#### Stair use

Stair use was assessed by placement of infrared beam sensors at select stairwell entry points at all participating worksites. The methodology was adapted from procedures developed by the Centers for Disease Control and Prevention for assessment of stair use in their facility [15]. Daily stair traffic counts were recorded by study staff for four consecutive weeks (20 working days) at baseline and 24 months, and for one week (five working days) at 12 months.

#### Health media environment

Presence of media (signs, posters, magazines, videos, etc.) related to eating, physical activity, or other health behaviors was recorded by study staff at baseline, 12, and 24 months using standardized forms.

### Participant measures

At baseline and 24 months, participants were invited by study evaluation specialists to take part in anthropometric visits scheduled at their respective worksites, during daytime business hours. Each site set aside a conference room or other enclosed area to ensure confidentiality during data collection. Informed consent was obtained at the start of the baseline visit. Each participant was given a uniquely identified, password-protected web survey link and instructions for completing an online study survey. Paper surveys were offered as an alternative to participants who did not wish to complete a survey online; requests for paper surveys represented 1.1% (*n *= 20) of all surveys distributed at baseline, and 0.8% (*n *= 11) of all surveys distributed at 24 months. Participants were compensated $10 for completion of each measurement visit and $10 for completion of each survey. Baseline evaluation of participants took place between January 2006 and April 2007; 24-month evaluation took place between January 2008 and March 2009. Evaluation team specialists, trained by the project director, conducted all participants' measurements and did not participate in intervention delivery activities. Measures reported here are as follows:

Height and weight. Height was measured to the nearest 0.1 cm by study staff at baseline using a portable stadiometer. Weight was measured to the nearest 0.1 kg by study staff at baseline and 24 months using a calibrated Seca 882 digital scale, with participants wearing light street clothes without shoes. BMI (kg/m^2^) was calculated from measured height and weight.

Demographic characteristics. Baseline survey questions assessed age, sex, race/ethnicity, marital status, educational attainment, smoking status, and pregnancy status.

### Intervention procedures

Blocked randomization at the worksite level (block size = 2), using computer-generated algorithms and performed by the study data manager, was used to assign worksites to intervention or no-contact control conditions after completion of baseline site evaluations and participant data collection within each site pair. Sites were recruited by the principal investigator and project manager and randomized by the data manager in the following pairs, according to time of study entry: two community colleges; a regional insurance office and a beauty industry corporate headquarters with an attached manufacturing and distribution center; and a utility company home office and a national headquarters for a health-related nonprofit organization. Site 1 intervention activities began in June 2006, Site 2 intervention began in October 2006, and Site 3 intervention began in May 2007; at all sites, intervention activities lasted for two years. There was no blinding of participants or study staff to assignment of worksites to intervention versus control condition. Control sites had no contact with study staff, except to engage in evaluation procedures at baseline, one, and two years; following the last round of data collection, control sites were offered a DVD containing intervention materials (e.g., poster templates, newsletter content, descriptions of intervention activity procedures) and an opportunity to ask questions of intervention staff as needed. Two of three control sites requested and received materials following study completion.

Intervention components were primarily targeted at making changes at the worksite level and are described as follows:

Food environment. The primary aims of the food environment intervention were: 1) to increase the availability of calorie smart foods (as defined earlier) to at least 50% of all cafeteria and vending machine offerings, as defined by previous work by colleagues in this area [13,26,27], 2) to reduce the price of calorie smart foods by 15% while increasing the price of non-calorie smart foods by 15%, 3) to offer smaller portion sizes as substitutes (e.g., 12 oz. soda cans to replace 20 oz. bottles in vending machines or cafeteria lines), and 4) to label calorie smart items at the point of purchase and promote these items through table tents in the cafeteria and posters near vending machines. Benchmarks for calorie smart food presence (i.e., 50% or greater) were determined from prior research [26,27] and were communicated to worksites by intervention staff, primarily the lead interventionist who had extensive food service experience, who worked directly with site food managers and vending delivery drivers to facilitate changes.

Physical activity environment. The primary aims of the activity environment intervention were to promote walking at work (via organized group walks, competition between co-workers, and activity monitoring) and to encourage stair use. Participants were provided with pedometers and access to a free online step tracking site http://www.americaonthemove.org for use throughout the intervention. Up to seven 6- to 8-week walking challenges were implemented, with input from study staff, at each intervention site. Walking challenges involved assisting worksite employees in organizing competitions designed to encourage walking as an activity to promote health. Employees were grouped into competitive teams (self-selected or based on worksite units) and tracked step counts collected during challenge periods; other walks were staged around charitable giving events or fun activities at work (e.g., games played outdoors while walking). In addition, regular walking was encouraged as a means of meeting activity goals during the workday (e.g., by promotion of walking meetings, taking time from lunch to walk, or walking before or after shifts). Motivational signs, decorative posters, and music were placed in select stairwells to enhance the stair environment and promote use.

Body weight tracking environment. Balance beam scales were placed at four accessible yet private locations (e.g., restroom or break room) at each intervention site. BMI charts were posted near scales to promote knowledge of parameters for healthy weight, and weight tracking forms were placed nearby to encourage participants to monitor their weight regularly. Up to three weight tracking competitions, framed around maintaining current weight (e.g., during the winter holidays) were held at each intervention site to encourage social support for weight tracking during the study.

Health media environment. In addition to placement of signs and posters related to food, activity, or body weight intervention targets, a two-page monthly newsletter was created by intervention staff and distributed for 24 months, via worksite email channels, at all intervention sites. The first page of the newsletter addressed general information related to healthy eating, activity, or other relevant behaviors; the sidebar on page one also presented site-specific information regarding upcoming events. The second page reported recent site-specific activities (e.g., competition results, co-worker testimonials).

Advisory panels. At each intervention site, advisory panels of 8-11 worksite employees were instituted to provide guidance and ongoing feedback to study staff. At each site, the worksite liaison identified during recruitment served on the panel and assisted with recommending additional site employees for the panel; efforts were made to ensure that the advisory panel represented a cross-section of employee classifications and organizational units. Panels met every other month during intervention to advise study staff on planning, implementation, and acceptability of all intervention activities.

### Statistical analysis

This study was designed as a group-randomized trial. All data were analyzed using mixed-model analysis of covariance (ANCOVA), assuming a Gaussian distribution of error terms [28]. Models were estimated in SAS 9.2 using the PROC MIXED command. Treatment condition (intervention vs. control) was modeled as a fixed effect, with worksite included as a random effect and individuals clustered within worksites. The primary outcome examined here was BMI change between baseline and follow-up; based on the known weight gain trajectory of free-living adults without intervention [3], the study was powered to detect a 1.5 kilogram difference in body weight between intervention vs. control sites at two years, accounting for approximately 15% attrition from worksites and adjusted for the worksite component of variance (80% power, *p *< 0.05, one-tailed). Models were adjusted for baseline BMI and known correlates of BMI, including age, educational attainment, and race/ethnicity [2]; we also adjusted for smoking status at baseline, as the percentage of smokers was significantly higher in control vs. intervention sites in this study, [χ^2^(1) = 4.14, *p *= 0.04]. All adjustment variables were analyzed as individual-level, fixed-effect covariates. Associated 95% confidence intervals and *p*-values were derived from *t*-statistics with 4 degrees of freedom, reflecting the group-randomized design.

After analyzing complete-sample data, models were stratified by sex to examine potential response differences for men versus women, in part due to greater tendency for women to participate in weight-related programs and activities relative to men [29]; 95% confidence intervals and *p*-values for the stratified models were based on *t*-statistics with 3 degrees of freedom. Primary study analyses include only individuals with complete data at both time points. Seventy-five women who were pregnant during at least one data collection point or who had missing data on pregnancy status for at least one time point were excluded from all analyses.

## Results

Table 2 reports baseline demographic characteristics by treatment assignment. Randomization achieved balance in background characteristics across sites; as reported in the Statistical Analysis section above, the percentage of smokers differed in control versus intervention sites (15.6% vs. 12.1%, *p *< 0.05). All other demographic comparisons between intervention and control sites were nonsignificant (*p *= 0.10 - 0.86). Mean baseline BMI was 28.4 kg/m^2^, which was nearly equal across sites, and 28.7 kg/m^2 ^at follow-up. At baseline, 68.8% of participants were either overweight or obese, and 70.9% were either overweight or obese at follow-up. Those lost to follow-up were not statistically different in terms of mean baseline BMI from participants who remained in the study (*p *= 0.47). Individuals in the study were comparable to the regional workforce in terms of age range, percent attaining a bachelor's degree at least, and race (white vs. not) [30]; participants were more likely to be women than the state population (60.5% vs. 50.4%), which is typical of weight-related trials [29], and were less likely to be smokers (13.9% vs. 16.1%) [31]. No adverse events were reported during the course of the trial.

**Table 2:** Demographic Characteristics of Participants at Baseline, by Treatment Assignment

	N (%) or Mean (SD)	Intervention % or Mean (SD)	Control % or Mean (SD)
**Total Number of Participants**	1,672	723	949
**Age**			
Under 30	275 (16.8%)	18.3	15.6
31-40	414 (25.3%)	24.3	26.0
41-50	517 (31.5%)	31.1	31.9
51-60	378 (23.1%)	23.4	22.8
Over 60	55 (3.3%)	3.0	3.7
**Sex**			
Women	1,011 (60.7%)	62.3	59.5
Men	654 (39.3%)	37.7	40.5
**Race/Ethnicity**			
Non-Hispanic White/Caucasian	1,429 (86.8%)	87.9	86.0
Non-Hispanic Black	70 (4.3%)	3.4	4.9
Other, Non-Hispanic	73 (4.4%)	4.6	4.3
Mulit-Racial, Non-Hispanic	22 (1.3%)	1.4	1.3
Hispanic	36 (2.2%)	2.0	2.4
Undefined/Refuse to Answer	16 (1.0%)	0.7	1.2
**Educational Attainment**			
Less than High School or High School Degree	156 (9.5%)	9.0	9.8
Technical Degree or Some College	497 (30.2%)	30.6	29.8
College Degree	615 (37.3%)	38.6	36.3
Graduate Degree	380 (23.0%)	21.8	24.0
**Marital Status**			
Never Married	277 (16.8%)	17.1	16.6
Married	1,001 (60.8%)	61.3	60.5
Cohabiting	119 (7.2%)	7.0	7.4
Separated	23 (1.4%)	1.8	1.1
Divorced	206 (12.5%)	11.5	13.3
Widowed	20 (1.2%)	1.3	1.2
**Smoking Status**			
Non-Smoker	1,411 (85.8%)	87.7	84.4
Current Smoker	233 (14.2%)	12.3	15.6
**Body Weight Status**			
Weight (kg)	81.5 (20.1)	82.2 (21.0)	81.1 (19.4)
BMI	28.4 (6.3)	28.7 (6.6)	28.3 (6.1)
Percent Overweight	35.8%	33.8%	37.3%
Percent Obese	33.0%	34.5%	31.9%

Table 3 presents a brief description and summary results for each intervention component. Within the dietary environment, the goals of increasing the proportion of healthy offerings in the cafeteria and vending machines to at least 50% of all available offerings were met at all intervention sites. However, the intervention goals of increasing prices of unhealthy foods by 15% and decreasing prices of healthy foods by 15% were not met at any of the intervention sites (data not shown).

**Table 3:** Description of HealthWorks Intervention and Summary of Two-Year Process Measures Findings

Intervention Component	Intervention Element	Goals	Process Results
**Dietary Environment**	Cafeteria Interventions	Change food offerings to bring overall product mix to > 50% calorie smart foods and beverages.	Goal achieved at all sites, with an overall mean of 53% of foods and beverages rated as calorie smart at 24 months, compared to 45.8% at baseline (50%, 53%, and 56% by site, compared to 49%, 43%, and 46% at baseline, respectively).
		Reduce price of calorie smart items by 15% and increase price of comparable non-calorie smart items by 15%.	Not successfully implemented at any of the intervention sites.
	Vending Machine Interventions	Change vending food offerings to bring overall product mix to > 50% calorie smart foods and beverages.	Goal achieved at 2 of 3 sites, with 63% and 68% of available vending machine foods and beverages rated as calorie smart at these sites at 24 months, compared to 40% and 45% at baseline, respectively. The remaining site achieved 48% calorie smart foods and beverages in vending machines at 24 months, compared to 35% at baseline.
		Reduce price of calorie smart items by 15% and increase price of comparable non-calorie smart items by 15%.	Not successfully implemented at any of the intervention sites.
**Physical Activity Environment**	Stairwell Enhancement Intervention	Increase stairwell use by enhancing stairwell attractiveness with art or inspirational posters, and point of entry signage.	Goal fully met at the 2 of 3 sites via music, art and film or motivational poster placement in stairwells, and signage at points of stair entry or at elevator vs. stair choice points. The remaining site complied only with placement of signage at elevator vs. stair choice points.
	Pedometer Intervention	Promote walking through pedometer use and participation in the 10,000 steps a day program and by offering up to 5 walking challenges and up to 4 combined activity/weighing challenges and facilitating the formation of ongoing walking clubs.	Component successfully delivered at all sites, which offered the recommended number of challenges during the study period.
**Body Weight Tracking Environment**	Scale Access Intervention	Promote regular self-weighing through placement of scales with BMI charts, weight lockboxes, and tracking forms at various locations in the worksite, as well as offering up to 3 weighing challenges.	Component successfully implemented at all three intervention sites, which each placed 4 scales and offered up to 3 weighing challenges and four combination weighing/activity challenges during the study period. Intervention site participants increased self-weighing frequency over time relative to control site participants (*p *< 0.0001).
**Health Media Environment**	Worksite Wide Publicity	Promote the intervention by posting healthy eating and activity information in multiple settings around the worksite.	Posters on walls and in display cases, table signs in the cafeteria, calorie signs in food lines, and signs on vending machines were successfully placed at all sites. Study staff achieved a 279.3% increase in media presence at intervention sites, relative to 142.2% increase in control sites (*p *< 0.10).
	Newsletters	Send monthly newsletters to promote positive behavioral messages and report news in the obesity field, progress data, and resource information.	Study staff successfully sent 24 monthly newsletters by email (pdf format) at all intervention sites.

For physical activity, the goal of enhancing stairwells was met in two of three sites; goals were met in all three sites for promotion of walking and pedometer use. For body weight awareness, the goal of placing four balance beam scales with accompanying information was met at all three sites. All participants reported weighing themselves at the same frequency per month at baseline [mean (SE) frequency: 5.39 (0.42) days for control vs. 5.59 (0.46) days for intervention, *p *= 0.77], though intervention participants significantly increased frequency of self-weighing on average relative to control participants [mean (SE) = 5.60 (0.52) days for control vs. 7.92 (0.55) days for intervention, *F*(1,4) = 9.36, *p *< 0.05].

The goals of publicizing the intervention around worksites (via wall or tabletop signs in cafeterias and signs adjacent to vending machines) and of disseminating a monthly newsletter for 24 months were met at all three sites. Presence of relevant media (e.g., nutrition information, physical activity promotion, or weight-related media) was highly variable; at baseline, study staff counted 3, 12, or 67 appearances of relevant media at intervention sites, versus 0, 5, or 45 appearances at control sites; at 24 months, counts ranged from 70, 106, and 135 at intervention sites versus 26, 36, and 47 at control sites. Presence of relevant media showed a nonsignificant mean increase of 76.3 (SD = 9.9) counts at intervention sites over the two-year study period, compared to a mean increase of 21.3 (SD = 11.2) at control sites [*t*(2) = 0.63, (*p *= 0.07)].

Table 4 presents the overall effect of the intervention on BMI and results stratified by sex. Using a mixed effect ANCOVA model, the adjusted mean weight gain at intervention sites was 0.32 kg/m^2^, versus 0.19 kg/m^2 ^at control sites, for an adjusted mean increase in BMI of 0.13 kg/m^2 ^(95% CI: -0.21, 0.46) units higher at intervention sites relative to controls; this difference was not statistically significant (*p *= 0.36). Percent overweight rose from 35.8% to 36.1% on average across all sites, and percent obese rose from 33% to 34.8%, with no difference between intervention versus control sites (data not shown). Additional sensitivity analyses in which those with extreme changes in BMI (± 10 units) were excluded had no meaningful impact on results. No significant differences by sex were observed.

**Table 4:** Effect of Intervention on Mean BMI Change over 24 Months

	Mean BMI Change: Intervention	Mean BMI Change: Control	Difference: Intervention - Control	95% Confidence Interval	*p*
**Total Sample**					
Unadjusted (*n *= 1,325)	0.30	0.19	0.12	-0.27, 0.51	0.46
Adjusted (*n *= 1,322)	0.32	0.19	0.13	-0.21, 0.46	0.36
**Women**					
Unadjusted (*n *= 823)	0.35	0.35	-0.01	-0.46, 0.44	0.97
Adjusted (*n *= 822)	0.35	0.35	0.01	-0.44, 0.46	0.97
**Men**					
Unadjusted (*n *= 502)	0.34	-0.10	0.45	-0.19, 1.09	0.11
Adjusted (*n *= 500)	0.36	-0.13	0.49	-0.19, 1.16	0.11

All models are mixed model ANCOVA models. Unadjusted models include baseline BMI, and adjusted models also include age, education, race, and smoking status; adjusted models for the Total Sample also include sex. Statistical tests for the total sample are based on 4 degrees of freedom, while tests for the models stratified by sex are based on 3 degrees of freedom

## Discussion

The primary hypothesis in this study was that a two-year, continuous multi-component intervention targeting environmental factors in worksites would result in less weight gain than no treatment. This hypothesis was not confirmed. Although a multi-component intervention effort was sustained over the desired length of time, with most of the elements of the intervention successfully implemented and weight change outcomes assessed in all study participants, weight gains over the course of the study did not differ between treatment and control worksites. Average weight gains in the cohort were approximately what would be expected in an untreated population sample over two years, about 1-2 kg [3], and the prevalence of obesity defined by BMI also increased correspondingly.

Results were comparable with another trial, conceptualized by colleagues in common with the present study and conducted concurrently, in which similar environmental changes were enacted with transit workers (a higher-risk population due to the sedentary nature of their employment) in the same metropolitan area as the present study; in that group-randomized trial with highly comparable methodology and intervention approaches, a nonsignificant intervention effect with a highly similar 95% confidence interval was observed [27]. Similarities between outcomes of these two parallel studies may be attributed to shared features of the study design, comparable approaches to intervention development (at the theoretical and implementation levels), and nonspecific, unmeasured features of the metropolitan area in which these trials were implemented. The present results were inconsistent with a quasi-experimental trial that saw better weight maintenance over two years in intervention versus control sites; however, that trial did not randomize at the site level and offered more intensive individual counseling and management-focused strategies to promote program offerings [24].

One possibility for the lack of an observed intervention effect on weight gain or obesity in this study is that the intervention was not strong enough to produce the desired effect on weight because of implementation failure. The failure to implement sustained changes in food prices was certainly a disappointment in this area, and the proximal cause of this failure is informative. Worksite directors were supportive of price changes in principle, but food service managers and vending drivers strongly resisted compliance due to concerns about possible adverse economic consequences, inconvenience, and competition with other vendors, despite all efforts to present evidence that these changes would not adversely affect profit margins or purchase of products [13]. Attributing the intervention failure to this component alone, however, seems to be an overstatement, as the remaining intervention goals for the study were largely met.

Intervention weaknesses, as well as variability between companies with regard to willingness to alter environmental characteristics (e.g., the third intervention site refused to enact most stairwell changes during intervention despite agreement to do so at the time of recruitment), may account for the inability to produce enough behavior change to have a measurable effect on body weight. The results of this study underscore that changing the environment in which people live is not an easy task. Aspects of the work environment may be constrained by the entrenched nature of workplace routines and policies, security needs, and economic considerations that are likely resistant to changes advocated by outside partners in particular. Enlisting the feedback of employee advisory committees might not have been enough to alter this entrenched worksite culture; future efforts should consider working more directly with worksite policymakers to enact changes company-wide with full input from these worksite partners [27].

An additional point worth considering, given that the current findings suggest limited effectiveness of this worksite weight gain prevention approach, is that the basic premise underlying workplace health promotion interventions may not be correct. Employed adults do spend approximately half of their waking hours at work, but the workplace may not be a major source of influences causing weight gain. Thus, even if changes in the worksite environment, such as improved food choices, are made, the proportion of the employee population affected by these changes may be rather small, suggesting a need to broaden or change the scope of environmental obesity intervention targets beyond that of the worksite.

This study was not without limitations. Foremost, whereas the trial was successful at achieving its recruitment goal of six worksites, this number represents only 3% of the sites that were initially contacted for participation, or 16% of sites that were eligible and expressed initial interest in the trial. Worksite contacts were straightforward about competing financial interests or goals of national corporate representatives, pending corporate transitions, or excessive time demands that precluded participation in the current trial; all of these factors were out of control of the investigators and reflect current economic demands on the workplace rather than outright recruitment failure within the trial itself. The group-randomized design limits power due to the small sample size at the group level, though we controlled statistically for the study design and were encouraged by high levels of individual responding to surveys across sites at baseline (mean baseline response rate = 71.4%, range = 64.6-77.6%) and excellent cohort retention among employees remaining at two year follow-up (mean two-year retention rate = 96.4%, range = 91.6-99.5%), which reflects positive engagement of trial staff with worksite employees.

## Conclusions

In sum, although evidence suggests that health promotion programs of all kinds in worksites are beneficial, it may be asking too much to expect such programs to have an effect on health outcomes in isolation. Recent reviews and commentaries on the idea that environmental changes are the key to rising obesity increasingly recognize that crude aspects of environments (e.g., proximity of unhealthy foods) are not universally associated with individual obesity risk [32]. This finding does not negate the premise that the rapid change in population obesity is an environmental phenomenon. However, simplistic notions about changes in such aspects of the environment may overstate the influence of physical features of the environment, which may be a consequence of other environmental features with the potential for greater causal influence (e.g., the social or media environment), rather than assuming physical environmental changes as primary causal drivers. In consideration of a social ecological framework, interventions may need to consider simultaneous changes at multiple levels to promote change. Promising directions for future research include promotion of greater engagement of relevant stakeholders in employee health and wellness, food service, and upper management, by combining environmental actions with more intensive individual dietary and physical activity counseling or incentivized individual approaches to worksite wellness [16,24,33], or by engaging household members [34] to enhance outcomes, as changes to the workplace environment may be necessary but not sufficient to change obesity-related health behaviors of individuals.

## Competing interests

The authors declare that they have no competing interests.

## Authors' contributions

JAL supervised the trial, contributed to analyses, and drafted the manuscript; KEN analyzed data and drafted a section of the manuscript; RFM supervised data analyses and helped to draft the manuscript; NRM contributed to analyses and helped to draft the manuscript; LJH participated trial development and implementation and helped to draft the manuscript; JMC participated in intervention delivery and helped to draft the manuscript; DJG contributed to analyses and helped to draft the manuscript; RWJ conceived of the study, secured funding, and drafted a section of the manuscript. All authors read and approved the final manuscript.
